# Case report: Outer lamellar macular hole and outer retinal detachment within myopic foveoschisis post-cataract surgery

**DOI:** 10.3389/fmed.2023.1154338

**Published:** 2023-04-17

**Authors:** Yii Hern Eng, Kah Wei Ong, Meng Hsien Yong, Wan Haslina Wan Abdul Halim, Mae-Lynn Catherine Bastion

**Affiliations:** Department of Ophthalmology, Faculty of Medicine, Universiti Kebangsaan Malaysia, Kuala Lumpur, Malaysia

**Keywords:** myopic foveoschisis, cataract, outer retinal detachment, outer lamellar macular hole, myopia

## Abstract

**Background:**

This study aimed to report a case of outer lamellar macular hole and outer retinal detachment within myopic foveoschisis (MF) post-cataract surgery.

**Case presentation:**

An elderly female patient with bilateral high myopia and pre-existing myopic foveoschisis underwent uncomplicated sequential cataract surgeries 2 weeks apart. She was able to achieve a satisfactory visual outcome for her left eye with stable myopic foveoschisis and visual acuity of 6/7.5, near vision N6. However, her right eye vision remained poor postoperatively, with a visual acuity of 6/60. Macular optical coherence tomography (OCT) revealed a new right eye outer lamellar macular hole (OLMH) and outer retinal detachment (ORD) within pre-existing myopic foveoschisis. Her vision remained poor after 3 weeks of conservative management, and she was offered vitreoretinal surgical intervention with pars plana vitrectomy, internal limiting membrane peeling, and gas tamponade. However, she refused surgical intervention, and her right vision remained stable at 6/60 over 3 months of follow-up.

**Conclusion:**

Outer lamellar macular hole and outer retinal detachment within myopic foveoschisis can occur soon after cataract surgery, which may be related to the progression of associated vitreomacular traction, and have a poor visual outcome if left untreated. Patients with high myopia should be informed of these complications as part of pre-operative counseling.

## 1. Introduction

Myopia is a complex ocular disorder with multifactorial etiology in which the optical power of the eye is too strong for the corresponding axial length. Pathological myopia is defined as high myopia associated with structural changes in the posterior segment, which can lead to potential sight-threatening complications, such as myopic foveoschisis, lacquer cracks, chorioretinal atrophy, and neovascularization (1).

High myopia is also associated with cataract, which tends to appear at an earlier age and progress rapidly. Patients with pre-existing macular conditions, such as myopic foveoschisis, might require cataract extraction to improve their vision. However, the surgical management of this group of patients poses a dilemma whether one should perform cataract surgery alone or in combination with vitrectomy. Lai et al. (2) reported that patients with myopic foveoschisis and cataracts may benefit from cataract surgery alone. Another study reported that cataract surgery alone appeared to increase the incidence of retinal detachment in the highly myopic eye (3). Hence, it is crucial to address patients' expectations and counsel patients regarding the potential complications during and after cataract surgery. In this case report, we wish to highlight a scarcely reported complication in patients with pre-existing myopic foveoschisis, namely outer lamellar macular hole and retinal detachment, after uncomplicated cataract surgery.

## 2. Case presentation

**Figure 1:**
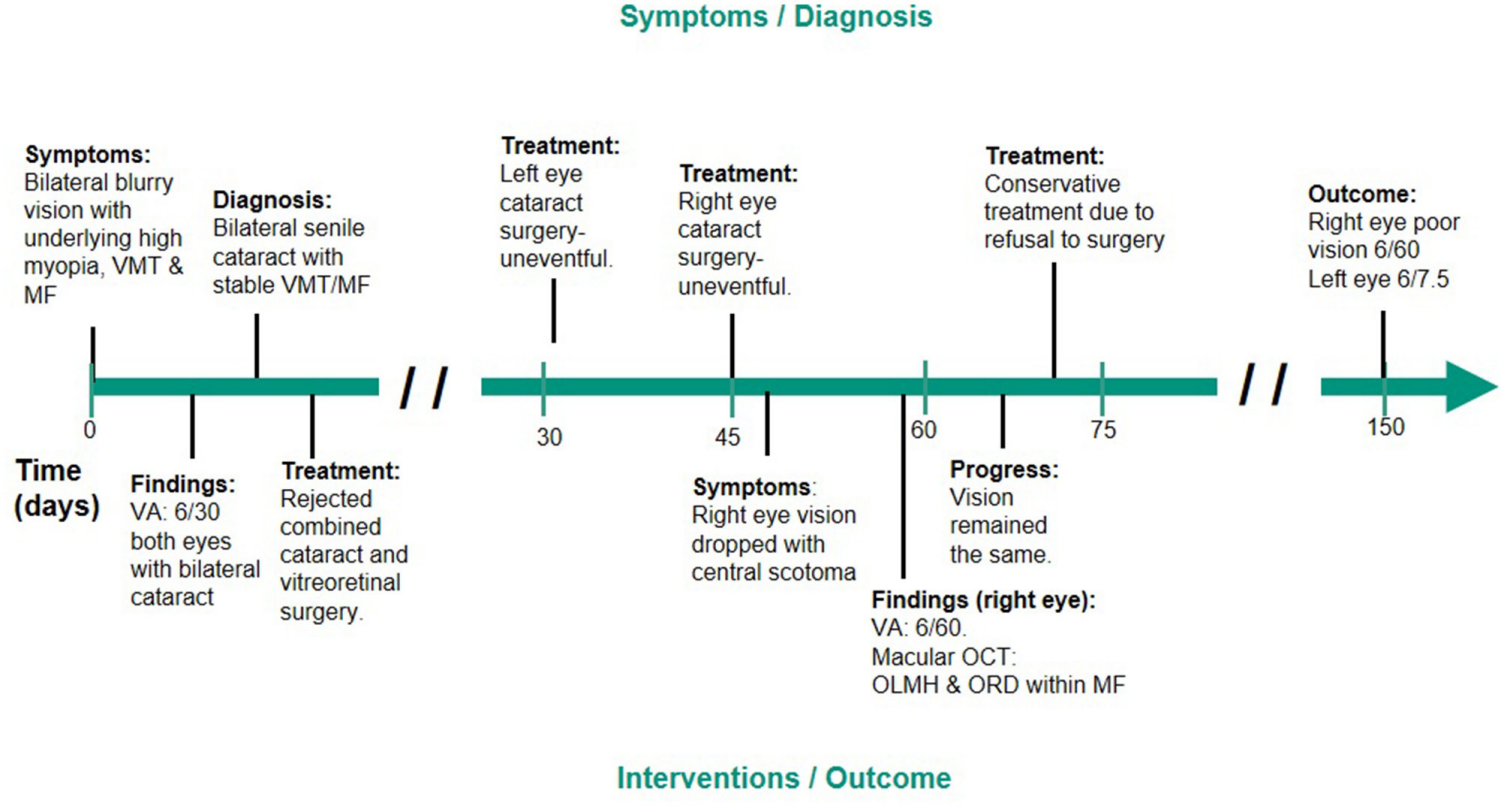
Clinical timeline—A 77-year-old woman with reduced visual acuity and central scotoma after her right eye cataract surgery. VMT, vitreomacular traction; MF, myopic foveoschisis; VA, visual acuity; ILM, internal limiting membrane; OCT, optical coherence tomography; OLMH, outer lamellar macular hole; ORD, outer retinal detachment.

**Figure 2:**
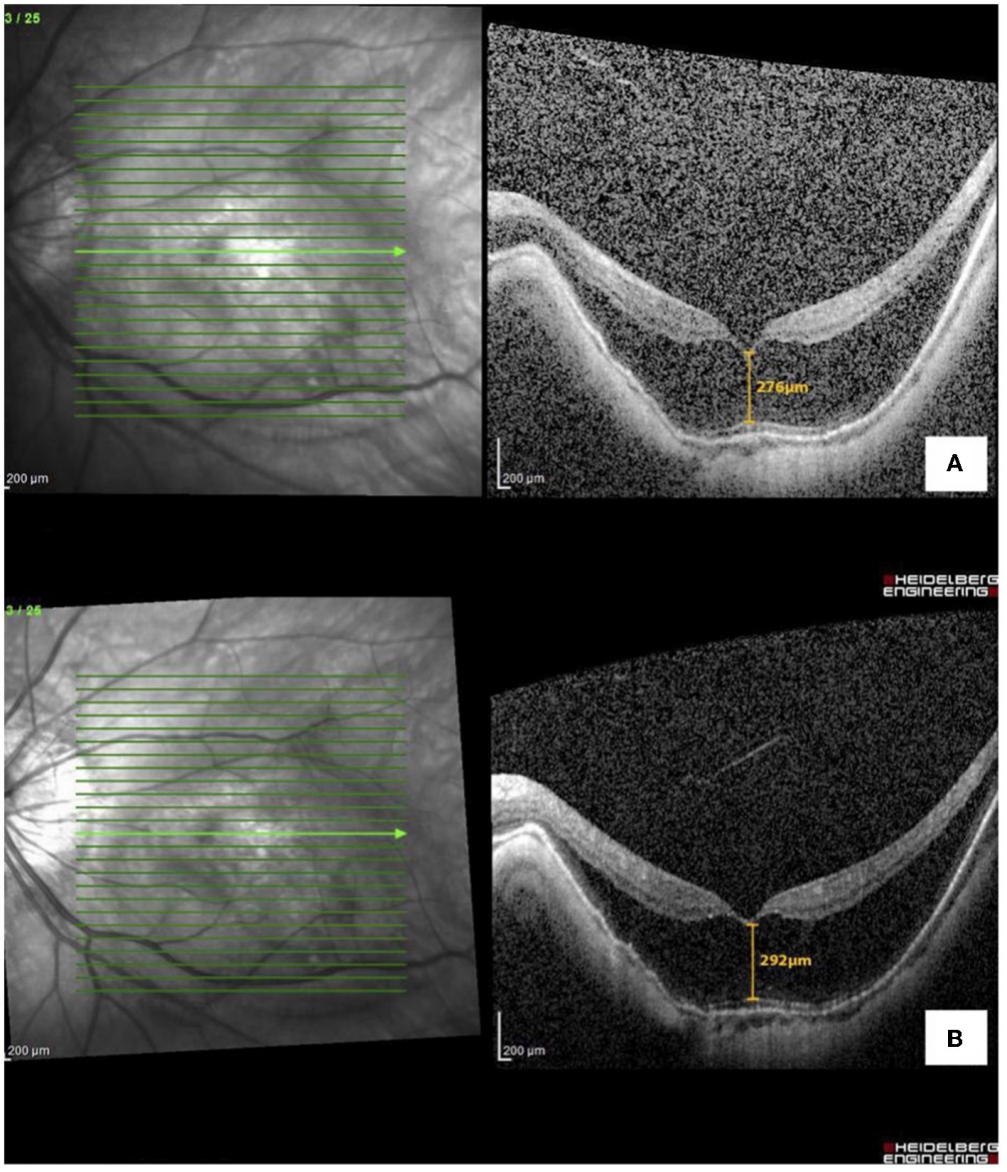
Optical coherence tomography macula of the left eye with **(A)** myopic foveoschisis pre-operatively and **(B)** stable myopic foveoschisis post-cataract surgery.

**Figure 3:**
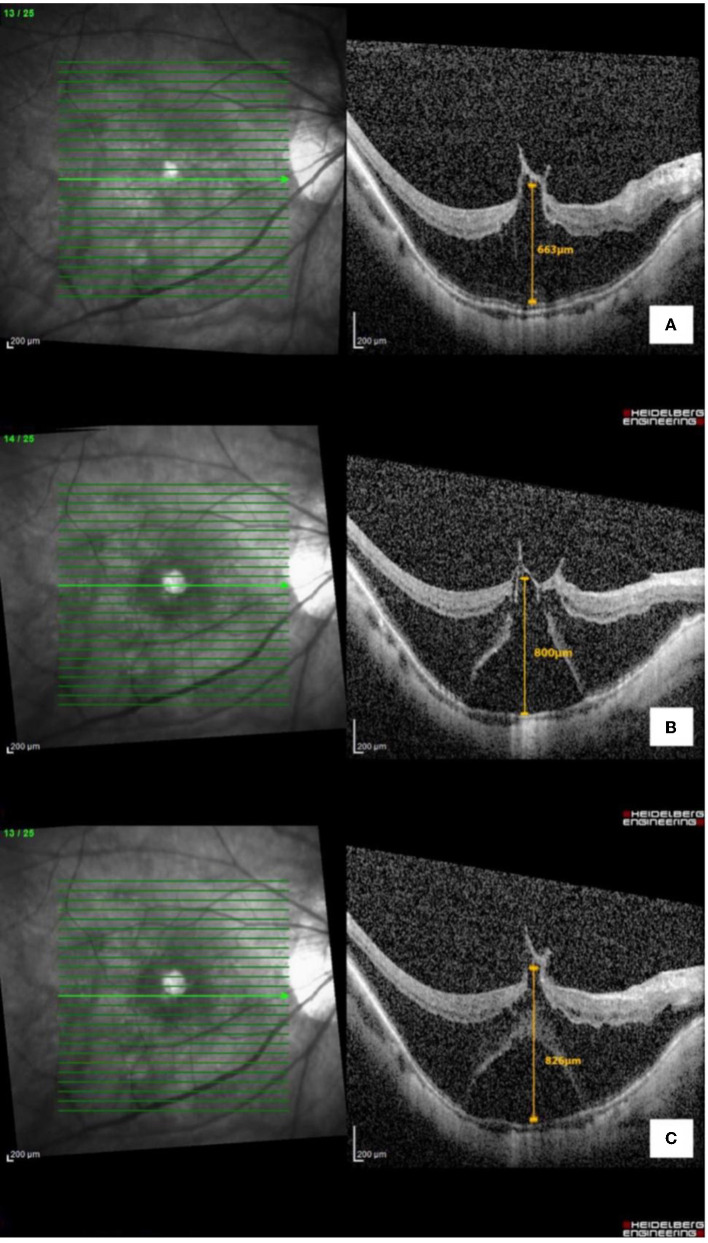
Optical coherence tomography macula of the right eye with **(A)** myopic foveoschisis with vitreomacular traction pre-operatively, **(B)** myopic foveoschisis with new-onset outer lamellar macular hole, and **(C)** macular detachment post-cataract surgery.

A 77-year-old woman with underlying diabetes mellitus had bilateral high myopia since childhood. Her axial length was 30.16 mm on the right eye and 31.02 mm on the left eye. She was on regular follow-ups for her pathological myopia with bilateral stable myopic foveoschisis for 4 years. During a recent follow-up, she complained of bilateral progressive blurring of vision with a visual acuity of 6/30. Figure 1 shows the clinical timeline for the patient. Ocular examination showed bilateral nuclear sclerosis cataracts with stable vitreomacular traction and myopic foveoschisis (Figures 2A, 3A). Combined cataract and vitreoretinal surgery were offered. However, the patient was not keen on vitreoretinal surgery. Consequently, the patient underwent sequential cataract surgeries 2 weeks apart. She had uneventful left eye surgery first with postoperative stable myopic foveoschisis (Figure 2B) on spectral domain optical coherence tomography (Heidelberg Spectralis Optical Coherence Tomography, Germany) and best-corrected visual acuity of 6/7.5, near vision N6. However, for her right eye, despite an uneventful cataract surgery, her vision remained at 6/60, with the recent onset of central scotoma 1 week after the surgery. Diagnosis of the outer lamellar macular hole (Figure 3B) and outer retinal detachment within her pre-existing myopic foveoschisis (Figure 3C) was confirmed with macular optical coherence tomography. Three weeks later, her right eye vision remained static, and she was offered vitrectomy with internal limiting membrane peeling and gas tamponade. However, the patient declined any surgical intervention due to her age and chose to be treated conservatively. Her vision was stable at 6/60 on subsequent assessment over 3 months of follow-up.

## 3. Discussion

### 3.1. Pathological myopia, foveoschisis, and macular hole

Pathological myopia is commonly associated with posterior staphyloma as its hallmark feature. It is defined as an outpouching of the wall of the eye that has a radius of less than the surrounding curvature of the wall of the eye (4). Myopes with posterior staphyloma are at significantly higher risk of developing myopic foveoschisis (5). The prevalence of myopic foveoschisis is reported to be 34% among myopic eyes (6), particularly in female patients (7), such as this patient, with varying clinical features which include schisis in the inner or outer retinal layers subsequently progressing into the macular hole or macular detachment. Myopic foveoschisis was first described by Takano and Kishi in 1999 as a degenerative retinal process within posterior staphyloma resulting in the splitting of the retinal layers. The pathogenesis of myopic foveoschisis involves complex anatomical mechanics induced by posterior ectasia, generating perpendicular traction on the scleral wall, choroid, and retina, as well as the tangential traction caused by the pre-macular vitreous cortex leading to the formation of macular hole (6).

Myopic foveoschisis is also considered one of the manifestations in the umbrella term of myopic traction maculopathy (MTM). Recently, Parolini et al. (8) have proposed a new MTM staging system (MSS) based on the OCT features of the retina. This classification system considers the retinal and foveal changes and tractional components seen on the OCT. Based on the MTM staging system, our patient's left eye remained stable at stage 2a (outer maculoschisis with a normal foveal profile). In contrast, her right eye progressed from stage 2b+ (outer maculoschisis with inner lamellar macular hole and epiretinal abnormality) to 3b+ (maculoschisis with detachment, outer lamellar macular hole, and epiretinal abnormality) post-cataract surgery. In this case report, we opted to use the term outer retinal detachment (ORD) within foveoschisis to explain the selective detached layer from the outer part of schisis, which is similar to the term maculoschisis–macular detachment in the MSS system.

### 3.2. Impact of cataract surgery on the progression of foveoschisis and macular hole

The mechanism postulated to expedite the progression of myopic foveoschisis in cataract surgeries is a disturbance to the vitreous body and an increase in vitreoretinal traction by the posterior hyaloid postoperatively. However, Cai et al. (9) argued that pre-operative vitreoretinal adhesion and longer axial length are the contributory factors in the progression of myopic foveoschisis while cataract surgery generally improves the visual acuity of patients with myopic foveoschisis. Our case justified the role of cataract surgery in patients with myopic foveoschisis, as the visual outcome was good in the left eye. However, an opposite outcome occurred in the fellow eye, despite uncomplicated cataract surgery. Furthermore, the axial length was shorter in the right eye. Pre-operative counseling plays a vital role in this situation whereby the progression of the myopic foveoschisis can occur regardless of the axial length.

The presence of vitreomacular traction is another factor to be considered before cataract surgery, which can progress to the macular hole or worsen the foveoschisis. In their case series, Mantopoulos et al. (10) showed that the effect of cataract extraction in eyes with pre-existing vitreomacular traction is varied. However, the study did not specifically study the myopic population, and the refractive status of patients was not documented. Our patient had obvious vitreomacular traction in her right eye before cataract surgery but refused to undergo combined vitrectomy with cataract extraction. Postoperative OCT revealed morphologically stable vitreomacular traction (Figure 2) in the vitreomacular interface. However, the degree of posterior hyaloid traction had changed, and this may contribute to the progression of foveoschisis and the development of the outer lamellar macular hole and detachment.

A previous study by Lai et al. (2) on the effects of cataract extraction for myopic foveoschisis showed stable foveoschisis and improved vision in their study population. Compared to our case, the baseline characteristics in the mentioned study are similar to the left eye of our patient. However, our patient's right eye had a much higher foveal thickness (663 μm) as compared to their study population (297 ± 107 μm). The study did not specifically mention the association of vitreomacular traction, as compared to the significant tractional component in the right eye of our patient. This again showed the importance of pre-operative counseling on this rare but possible complication, in patients with pre-existing myopic foveoschisis, especially in extremely high foveal thickness and cases associated with vitreomacular traction.

### 3.3. Management of retinal detachment in foveoschisis

There was no consensus on the definitive management of myopic foveoschisis. Polito et al. (11) observed spontaneous resolution of foveoschisis with retinal detachment after 4 months, which suggests the role of conservative management in myopic foveoschisis. The principle of surgical management of myopic foveoschisis focuses on relieving any identifiable retinal perpendicular and tangential tractional forces to produce retinal anatomical and functional improvement. Many vitreoretinal surgery modalities have been reported, and a combination of pars plana vitrectomy, internal limiting membrane peeling, with or without macular buckle, is generally believed to achieve a good visual outcome by eliminating both tangential and anterior–posterior tractions (12). Vitrectomy alone without inner limiting membrane peeling, but with careful removal of the posterior hyaloid, has also been shown to produce a comparable outcome compared with vitrectomy with inner limiting membrane peeling (13). Zhao et al. (14) demonstrated favorable long-term results of macular buckling for highly myopic eyes with both myopic foveoschisis and macular holes. However, in this case, the patient refused surgical intervention. The challenges of face-down posturing in the elderly patient may have been a deterrent in this case for the surgery.

## 4. Conclusion

Outer lamellar macular hole and outer retinal detachment within myopic foveoschisis can occur soon after cataract surgery, which may be related to the progression of associated vitreomacular traction. This may adversely affect the visual outcome after surgery. This group of patients should be informed of these complications as part of pre-operative counseling.

## Data availability statement

The original contributions presented in the study are included in the article/supplementary material, further inquiries can be directed to the corresponding author.

## Ethics statement

Ethical review and approval was not required for the study on human participants in accordance with the local legislation and institutional requirements. Written informed consent from the (patients/ participants OR patients/participants legal guardian/next of kin) was not required to participate in this study in accordance with the national legislation and the institutional requirements. Written informed consent was obtained for identifiable health information included in this case report.

## Author contributions

MY and YE: conceptualization. YE and KO: data curation and writing—original draft. MY: formal analysis, reviewing, and editing. MY, YE, and KO: investigation and project administration. MY and WW: methodology. MY, M-LB, and WW: supervision. All authors contributed to the article and approved the submitted version.
